# Students' and teachers' perceptions of clinical assessment program: A qualitative study in a PBL curriculum

**DOI:** 10.1186/1756-0500-2-263

**Published:** 2009-12-23

**Authors:** Hanan MF Al Kadri, Mohamed S Al-Moamary, Cees van der Vleuten

**Affiliations:** 1 Clinical Affairs Department. College of Medicine, King Saud bin Abdulaziz University for Health Sciences, Riyadh, Saudi Arabia; 2 Department of Educational Development and research. Faculty of Health, Medicine and Life Sciences. University of Maastricht, the Netherlands

## Abstract

**Background:**

"Examinations drive students' learning." This statement refers to what is assumed to be one of the strongest relationships in education. We explored in this research how and why students differ in their approaches to learning, how assessment affects deep learning, and which barriers stand in the way of good assessment and learning in the clinical years of a Problem Based Learning (PBL) graduate entry medical curriculum.

**Findings:**

**Method: **We conducted a qualitative, phenomenological study using semi-structured group interviews with students and semi-structured individual interviews with teachers and students. The transcripts were analyzed, and themes were identified.

**Setting**: The research was conducted at the King Saud bin Abdulaziz University for Health Sciences, College of Medicine, Riyadh, Saudi Arabia from November 2007 to March 2008.

**Results: **A total of 28 students participated in 7 focus group interviews. Semi-structured individual interviews were conducted with 12 teachers and 12 students. The analysis yielded four themes: summative assessment, formative assessment, continuous assessment of clinical attachments, and learning objectives.

**Conclusions:**

The results of this study confirm that assessment affects students' perceptions of learning and how they learn. These effects are not uniformly positive. According to the students, the predominantly summative assessment program offers little inducement to engage in deep learning. They express a clear preference for formative assessment, which may foster a deeper approach to learning. Efforts to achieve more clinically relevant assessment with adequate balance between the various types of assessment are required. Research is needed to decide this balance.

## Introduction

The concept that assessment drives learning has been accepted as one of the principles of good assessment practice [1]. Assessment affects not only what students learn but also how they learn [2]. Unfortunately, some student learning strategies contribute little to the learning processes intended by the curriculum [2].

The relationship between assessment and the learning behaviors of medical students has been examined by several authors[3,4]. In examining this relationship, Biggs focused on the concept of constructive alignment [5], advocating an approach to curriculum development that ensures that both teaching and assessment are aligned with the curriculum's stated learning objectives.

Different effects of assessment on learning have been reported in the literature. Some studies have shown that students' approaches to learning and their retention of knowledge differ across a range of assessment methods [4,6]. In a quantitative study, Gijbels et al [7] found a relationship between the learning environment, assessment demands as perceived by students, and students' approaches to learning; however, these findings require further clarification. A study by Baeten et al [8] found no evidence that assessment advanced a deep rather than a superficial approach to learning. Mattick et al [9] emphasized the importance of understanding the barriers to a deep learning approach as perceived by students.

In summary, if we want to enhance the students' learning and promote deep learning, we need to understand how and why students differ in their approaches to learning, how assessment affects deep learning, and which barriers stand in the way of good assessment and learning. In this study, we explored these issues by seeking students' and teachers' perceptions of their experiences with the assessment program in the clinical years of a PBL curriculum.

## Methods

We conducted a phenomenological study to examine what happens when students direct their study strategies to fit with assessment rather than learning objectives. Our aim was to gain insight into how students and teachers experienced the assessment program. We explored students' perceptions in semi-structured group interviews. When more depth into students' perceptions was required, we conducted semi-structured individual interviews with students until we reached data saturation. Semi-structured individual interviews were also performed to explore the perceptions of teachers.

### Study setting

The study was conducted at King Saud Bin Abdulaziz University for Health Sciences (KSAU-HS), College of Medicine (COM), Riyadh, Saudi Arabia in the period from November 2007 to March 2008. The college accepts only male students and is housed within King Abdulaziz Medical City, a 900-bed tertiary care center. The KSAU-HS, COM curriculum is a Problem-Based Learning (PBL) curriculum. It is a four-year graduate entry program consisting of two preclinical years and two years of clinical education. During the clinical years, there are two concurrent but different paths of learning, one of workplace learning and one of PBL group discussions.

### Assessment Program

**Figure 1:**
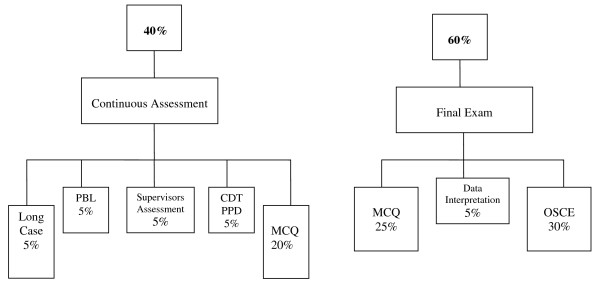
**Flow Chart of the Assessment Program in Years 3 and 4 at KSAU-HS, COM**. MCQ: multiple choice question. PBL: problem-based learning. CDT: community doctor theme. PPD: personal professional development. OSCE: objective structured clinical examination.

The assessment program for the clinical years at KSAU-HS, COM is block-based (total of five blocks). In each block, students' assessment is divided into two main parts (Figure 1). Assessment of students' performance during each block clinical attachments accounts for 40% of the final grade (students portfolio), and the final examination accounts for the other 60%. By the end of each clinical attachment (every 1-4 weeks), students meet with their clinical supervisors and are expected to receive a written qualitative formative assessment and feed back on their performance during that attachment. In the present paper the term "summative assessment" refers to an assessment performed to assign students a course grade, "formative assessment" refers to an assessment as an educational tool to aid students' learning without grading, and "continuous assessment" refers to an assessment of students' progress based on work they do or tests they take throughout the block.

### Study population

Students were recruited by a stepwise purposeful sampling approach. 56 out of a total of 61 students who had experienced the assessment program of the clinical years were invited to participate in the study. 28 students participated in seven semi-structured focused group interviews. Each group interview was attended by four students. Purposeful sampling was also used to select 12 teachers out of the pool of clinical years block directors and clinical supervisors. All teachers who were contacted agreed to participate and were interviewed individually to allow more freedom and avoid bias. To explore the obtained themes, 12 additional students out of the same pool were individually interviewed. All interviews were done by the principal investigator, who was at a comparable seniority with the interviewed teachers and had not yet started teaching any of the interviewed students at the time of data collection.

### Data collection

Students were asked to talk about their perceptions of the assessments and how their experiences with the assessment program affected their learning activities and strategies for handling assessments. Each group interview lasted about 40-75 minutes. We benefited from some of the conflicting opinions that were expressed by the students. When no new themes arose, we stopped conducting interviews. The interviews were recorded on tape, and students' nonverbal behavior was registered. The audiotapes were transcribed verbatim, and field notes and the verbatim transcriptions of the group interviews were integrated. This process was repeated for the individual semi-structured interviews with the students (12 students took part in interviews lasting 30-45 minutes) and teachers (12 teachers took part in interviews lasting 30-45 minutes).

### Data analysis

The transcriptions of the interviews were analyzed using Atlas-ti (Version 5.2) computer software. Analysis involved line-by-line scrutiny of the transcript and assignment of keywords to text fragments. For each interview, categories and themes were identified; these categories and themes were subsequently tested and refined in a cyclic analytical process, moving backward and forward between the interviews. This type of analysis resembles the open coding and axial coding phases of grounded theory [10]. This approach was chosen for its ability to provide systematic inductive guidelines for collecting and analyzing data. To capture a more complete and contextualized picture of the data, we performed investigators' triangulation by having a co-investigator code two interviews independently. There was minor disagreement, which was resolved by discussion. Furthermore, we improved the credibility and transferability of the data by member checking. The results were presented to a group of the students who were asked to give feedback. A similar procedure was used with the interviewed teachers. KSAU-HS ethics approval was obtained prior to conducting the research.

## Results

We invited 56 from a total of 61 clinical year students to participate, and 28 of them agreed to do so. Each student took part in one of seven focus group interviews, with four students per group. The students' mean age was 26.74 years, and their mean graduation GPA was 3.96/5. These characteristics are similar to the mean age (26.77 years) and the mean GPA at graduation (3.89/5) of the non-participating students. Twelve teachers were approached, and they all agreed to take part in the study.

The first analysis of the data generated four themes with its related codes (Table 1). We noticed diversity in the level of abstraction and aggregation of codes within themes and between students and teachers. Twelve additional individual semi-structured student interviews were performed for a better understanding of the obtained themes. In order to illustrate how our concept of the effect of assessment on student learning is grounded in the data, we present quotes from the transcripts.

**Table 1:** Themes within the coding system for both student and teacher interviews

	Theme	Description	Codes
1	Summative assessment	Codes on the effect of summative assessment on students' learning	Provoking anxiety; stressful; sporadic reading; has no role; a waste of time; unfair; summative is mandatory and summative is fair

2	Formative assessment	Codes on the effect of formative assessment on students' learning. Codes on the role of educational culture in students' learning and teachers' assessments	Improve students' learning; affected by culture; time consuming; should not be overdone; fair; learning strategy improvement and problem identification. Formative assessment affected by culture; changing educational culture

3	Clinical attachment assessment (continuous assessment)	Codes on the effect of continuous assessment and its weight on students' learning	Should be given more weight; fair

4	Objectives	Codes on the effect of objective-directed assessment and constructive alignment on students' learning	Heavy; fair; content coverage; blueprinting; assessment weight

### Students' and teachers' perceptions of the assessment program

#### Summative versus formative assessment

A majority of the students preferred summative exams as a discriminating factor between them, but they did not agree upon its effect on their future performance: "*I would prefer summative assessment because I will have evidence to compare myself with others*," and "*I don't think it will reflects whether the students are good or bad*." Some of the students were doubtful of the role of summative assessment in graduating better practicing doctors: "*Student who had a full grade might not be able to deal with patients*." On the other hand, a few students were not able to decide the superiority of summative or formative assessment role in their learning: "*It's confusing whether to choose summative or formative assessment,; each one has advantages and disadvantages*." Several students said that summative assessment caused anxiety: "*one hour of exam will determine your future; it is stressful*." This stressful situation makes some students resort to sporadic, patchy reading through hunting the information they think is important or might come in their exam. Some started even to neglect thinking about the exam mark and focused only on passing it aiming to avoid this stressful feeling: "*we have to read for the exam, we have to read for the clinical attachments; how can I get time in between, I am always stressed, very stressed." *And "*I don't care about the marks. If I care that much I'll be anxious and commit more mistakes*..". Summative assessment was also considered by some students as unfair, it didn't reflect the effort they put and the activities they do across the block. Therefore some students were regretting the time they spent in preparing and performing these exams: *"The exam is unfair.... I feel I'm wasting my time for nothing." *In reaction to these complex opinions, some students became marks hunters practicing several study strategies aiming for passing the exam or scoring high marks. Of these strategies was holistic reading or on the contrary strategic, selective reading: "*I will make sure that I could retain at least the minimum requirement*," and "*I read the important subjects like life threatening and emergency situations*." Some students studied based on their own selected objectives, preferences, or type of exam. They used their own opinion, exam experiences, feelings and speculation in creating their hidden curriculum: "*to pass the exam*... *I really need to remember some numbers, some percentages and things that can come in MCQ questions*" and "*I know some things about him or her that would make the examination easy for both of us*.".

A majority of the students clearly preferred formative assessment due to its attached feedback. This feedback helped them to identify their learning objectives and improve their study strategies. The students' views contradicted the opinions expressed by some of the teachers, who stated that summative assessment was an essential step in students' assessment, leading to fair assessment. Teachers did not deny the importance of formative assessment. However they thought that it was time consuming. If formative assessment is further increased, they may not be able to provide and maintain its performance; hence, it would be a waste of time. Interestingly, the expression "waste of time" was used by both students and teachers but with reference to different types of assessment. The teachers took into consideration the effect of the college's education culture on the implementation of formative assessment. "*In our education culture, we focus on the exam and how much we achieve on the exam."*. Both students and their teachers are mainly graduates of traditional curriculum where formative assessment was not part of their assessment methods. They thought that gradual adaptation to formative assessment and orientation of the learners and teachers to the process and to the goal of the process are important. Adequate time allowance recognized as essential prior to heavy formative assessment implementation.

#### Continuous attachment-related assessment

Assessments during clinical attachments (continuous assessment) were the composite of students' portfolios and were preferred by the students. It helped them improving their knowledge, and increasing self-awareness:*"Continuous assessment actually encourages the student to read more and to keep up to the maximum level that is required from him*.". The students felt that the weight placed on continuous assessment is very small. They emphasized that it should be sufficient enough to stimulate their clinical learning. Most of the teachers agreed with the students on the importance of clinical attachment assessment leading to better training: *"...More weight for the Continuous Assessment, students will give more attention to the clinical attachment."*

#### Objectives

An assessment program that reflects the curriculum's objectives was considered to be fair and reliable. It will enable them to correctly direct their reading based on what is planned for them in the curriculum.: *"I follow the objectives when I think about the exam."*. Therefore students think that an assessment program should not be standardized across the blocks and should be individualized based on each block objectives. What was more important to the students is that their supervisors should be familiar with the curriculum objectives. The teachers noted various problems in relation to the objectives, indicating that these objectives should be simple and easily implemented. It should not be broad and the depth of knowledge and skills required should be specified. In their view, fair assessment was characterized by a strong alignment with curriculum objectives. Teachers stressed the importance of 'blueprinting' and 'assessment weight' in designing fair assessments: *"A final exam should reflect all the curriculum components."*.

## Discussion

In this research, KSAU-HS, COM was taken as an example of a college that implement assessment program characterized by being mainly summative. The performed work was exploratory rather than definitive: its main value is to guide development of further enquiries and understanding. We kept in mind that even the best designed interventions will not always result in better learning for all students but for sure it will improve it at least for some.

In this study, the participating students said that they adapted their study strategies to task demands. The type of assessment and the weight accorded to it were significant factors that affected their approach to learning. This result is in agreement with other research, where assessment type [11] and weight [12] were identified as factors that influence students' approaches to learning.

The teachers in this study see more advantages of summative assessment than the students, with the latter group experiencing summative assessment as stressful, anxiety-provoking, and inducing sporadic and superficial reading. In contrast to the teachers, the students express a preference for formative assessment with feedback. Formative assessment known to produce greater increase in students' achievement than class size reduction or increase in teachers' content knowledge [13]. In fact even summative tests can provide ways of eliciting evidence of student achievement. If used appropriately, can prompt feedback that moves learning forward [14]. However using grades alone for feedback is found to be the poorest type of feedback [15].

Formative assessment used in the presented research is considered as medium cycle formative assessment (every 1-4 weeks). The existing research base shows that short-and medium-cycle formative assessments improve student achievement [16]. Having the students recognised the importance of this method of assessment despite its limited portion in the assessment program may be considered an extra evidence of its effectiveness.

The literature provides support mostly for the students' preferences, with a meta-analysis reporting that feedback produces the most powerful single effect on achievement [17]. The information processing needed for deep learning may be hampered when students do not spontaneously engage in cognitive activities that foster such learning [18]. Research supports the use of learning materials and teaching methods that encourage students to employ deeper learning strategies whenever possible [19]. To achieve this goal, the infrastructure needed to implement formative exams with appropriate accompanying feedback should be assessed. Formative feedback time, orientation of the learner to the process and to the goal of the process[20] are essential. Ruston [21] has discussed the presence of cultural difficulties to implement formative assessment and feedback. He questioned if the paradigm shift in assessment culture has occurred, as the majority of the existing literature is centered on summative assessment. The decision to accept and use formative feedback is influenced by several external and internal factors includes self-perceptions, emotion, reflection and professional culture [22]. Through using historical data, it was clearly demonstrated how student perceptions have changed over time as a result of internal and external influences [23].

The students' remarks that summative assessment provokes anxiety and stress give pause for thought. The primary sources of stress have been repeatedly found to be examination and grades [24], therefore care should be directed to students' perceived feelings. The problem is not solely related to the introduction of summative versus formative exams; it is wider than this view. Balancing formative and summative assessments is part of the work needed to reduce student anxiety. To what extent formative assessment should be used at the expense of summative assessment is an area that needs further investigation. The studied group was in their clinical years, where PBL sessions were parallel to their work-based learning. The cognitive and emotional effects of small group learning in PBL are not clear enough in the literature [25].

Anxiety in general affects the performance of any student. Joels [26] reported that stress within the context of a learning experience induces focused attention and improves remembering relevant information. Therefore, eliminating educational stress appears to be an impossible, unnecessary goal, whereas reducing unnecessary stress and improving the work environment should be targeted.

Both the students and teachers preferred continuous attachment-related assessment. The main benefits of portfolio use were improvements in students' knowledge and understanding, increased self-awareness, engagement in reflection, and improved student-teacher relationships [27]. However, students' and teachers' time commitment required for portfolio completion and evaluation may be a major drawback even if a portfolio was required as part of students' assessment. The assessors should be well prepared, 'trained' as assessors, and perceived to be fair, competent, skilful, and knowledgeable [28]. Therefore, adding the portfolio to the assessment program without appropriate infrastructure of faculty training and time allowance may contribute negatively to student anxiety and the educational culture.

The first priority in designing assessment program is to serve the purpose of promoting students' learning without forgetting the purpose of accountability, ranking and certifying competence. Assessment program that cover all the knowledge and competencies required based on Miller pyramids [29] is needed. While methods like MCQ, essay and oral exam can cover the knows and knows how levels. There is a need to implement types of work based assessment such as mini clinical examination (mini CEX), directed observation of practical skills (DOPS), long and short cases and others to test the students capabilities and competencies in both vivo and vitro. These should be accompanied by direct observation and feedback to allow improvement and guarantee competency.

Overall, a careful balance between formative, summative, and continuous assessment (portfolio) needs to be attained. To decide what is appropriate balance, further researches are needed.

Students and teachers agree that an assessment program should be aligned with the curriculum objectives. Learning models that are guided by curriculum objectives are effective educational tools that help students achieve a broad and direct exposure to core educational concepts [30]. Such a model will prevent the establishment of hidden curriculum, feelings of unfairness, and stress whenever an exam is conducted. Hence, it may stimulate a deep approach to learning.

Some weaknesses of this study should be mentioned. The population consisted entirely of male students. We are not aware of previous comparisons between male and female students regarding their perceptions of exams, but a gender effect cannot be excluded. This study has discussed students' perceptions of their ability to learn. Further work on the effect of assessment on student learning is needed. Finally, the focus on one medical college maybe considered as narrow. However, we believe that the research impact is broad. The research has evaluated the assessment program taking into consideration the instructional design, curriculum used while reflecting on the other published research on students assessment.

## Conclusion

The results of this study confirm that assessment affects students' perceptions of learning and how they learn. These effects are not uniformly positive. According to the students in this study, the predominantly summative assessment program offers little inducement to engage in deep learning. They express a clear preference for formative and continuous assessment, which may foster a deeper approach to learning.

It is important to be aware of the differing views held by students and teachers concerning the same educational assessment program. Faculty improvement programs and faculty time are important infrastructures for formative assessment implementation. Finally, efforts to achieve more clinically relevant assessment with adequate balance between the various types of assessment are required. Research is needed to decide this balance.

## Competing interests

The authors declare that they have no competing interests.

## Authors' contributions

HK conceived and wrote the research proposal, conducted the interviews, analyzed the data, and wrote the final manuscript. MM shared in the recruitment of students and teachers, conducted the first two interviews together with the primary investigator and analyzed them independently. He reviewed and approved the final manuscript. CV supervised the writing of the research proposal, read and commented on all the research steps, reviewed, corrected and approved the final manuscript.
